# The Power of Cut-Based Parameters for Computing Edge-Disjoint Paths

**DOI:** 10.1007/s00453-020-00772-w

**Published:** 2020-10-21

**Authors:** Robert Ganian, Sebastian Ordyniak

**Affiliations:** 1 Algorithms and Complexity Group, Vienna University of Technology, Vienna, Austria; 2 Algorithms Group, University of Sheffield, Sheffield, UK

**Keywords:** Edge-disjoint path problem, Feedback edge set, Tree-cut width, Parameterized complexity

## Abstract

This paper revisits the classical edge-disjoint paths (EDP) problem, where one is given an undirected graph *G* and a set of terminal pairs *P* and asks whether *G* contains a set of pairwise edge-disjoint paths connecting every terminal pair in *P*. Our aim is to identify structural properties (parameters) of graphs which allow the efficient solution of EDP without restricting the placement of terminals in *P* in any way. In this setting, EDP is known to remain NP-hard even on extremely restricted graph classes, such as graphs with a vertex cover of size 3. We present three results which use edge-separator based parameters to chart new islands of tractability in the complexity landscape of EDP. Our first and main result utilizes the fairly recent structural parameter tree-cut width (a parameter with fundamental ties to graph immersions and graph cuts): we obtain a polynomial-time algorithm for EDP on every graph class of bounded tree-cut width. Our second result shows that EDP parameterized by tree-cut width is unlikely to be fixed-parameter tractable. Our final, third result is a polynomial kernel for EDP parameterized by the size of a minimum feedback edge set in the graph.

## Introduction

Edge-Disjoint Paths (EDP) is a fundamental routing graph problem: we are given a graph *G* and a set *P* containing pairs of vertices (*terminals*), and are asked to decide whether there is a set of |*P*| pairwise edge-disjoint paths in *G* connecting each pair in *P*. Similarly to its counterpart, the Vertex-Disjoint Paths (VDP) problem, EDP has been at the center of numerous results in structural graph theory, approximation algorithms, and parameterized algorithms [2, 8, 9, 14, 17, 19, 21, 22, 26].

Both EDP and VDP are NP-complete in general [16], and a significant amount of research has focused on identifying structural properties which make these problems tractable. For instance, Robertson and Seymour’s seminal work in the Graph Minors project [22] provides an $\mathcal {O}(n^3)$ time algorithm for both problems for every fixed value of |*P*|. Such results are often viewed through the more refined lens of the *parameterized complexity* paradigm [5, 7]; there, each problem is associated with a numerical parameter *k* (capturing some structural property of the instance), and the goal is to obtain algorithms which are efficient when the parameter is small. Ideally, the aim is then to obtain so-called *fixed-parameter* algorithms for the problem, i.e., algorithms which run in time $f(k)\cdot n^{\mathcal {O}(1)}$ where *f* is a computable function and *n* the input size; the aforementioned result of Robertson and Seymour is hence an example of a fixed-parameter algorithm where $k=|P|$, and we say that the problem is FPT(w.r.t. this particular parameterization). In cases where fixed-parameter algorithms are unlikely to exist, one can instead aim for so-called XP algorithms, i.e., algorithms which run in polynomial time for every fixed value of *k*.

Naturally, one prominent question that arises is whether we can use the structure of the input graph itself (captured via a *structural parameter*) to solve EDP and VDP. Here, we find a stark contrast in the difficulty between these two, otherwise closely related, problems. Indeed, while VDP is known to be FPT with respect to the well-established structural parameter *treewidth* [24], EDP is NP-hard even on graphs of treewidth 3 [9]. What’s worse, the same reduction shows that EDP remains NP-hard even on graphs with a vertex cover of size 3 [9], which rules out fixed-parameter and XP algorithms for the vast majority of studied graph parameters (including, e.g., *treedepth* and the *size of a minimum feedback vertex set*).

We note that previous research on the problem has found ways of circumventing these negative results by imposing additional restrictions. Zhou et al. [26] introduced the notion of an augmented graph, which contains information about how terminal pairs need to be connected, and used the treewidth of this graph to solve EDP. Recent work [13], which primarily focused on the complexity of EDP on near-forests and with respect to parameterizations of the augmented graphs, has also observed that EDP admits a fixed-parameter algorithm when parameterized by treewidth and the maximum degree of the graph.

*Our Contribution* The aim of this paper is to provide new algorithms and matching lower bounds for solving the Edge-Disjoint Paths problem *without imposing any restrictions on the number and placement of terminals*. In other words, our aim is to be able to identify structural properties of the graph which guarantee tractability of the problem without knowing any information about the placement of terminals. The only positive result known so far in this setting requires us to restrict the degree of the input graph; however, in the bounded-degree setting there is a simple treewidth-preserving reduction from EDP to VDP (see Proposition 1), and so the problem only becomes truly interesting when the input graphs can contain vertices of higher degree.

Our main result, which is provided in Theorem 2, is an XP algorithm for EDP when parameterized by the structural parameter *tree-cut width* [20, 25]. Tree-Cut width is inherently tied to the theory of graph immersions; in particular, it has a similar relationship to graph immersions and cuts as treewidth has to graph minors and separators. Since its introduction, tree-cut width has been successfully used to obtain fixed-parameter algorithms for problems which are W[1]-hard w.r.t. treewidth [11, 12]; however, this is the first time that it has been used to obtain an algorithm for a problem that is NP-hard on graphs of bounded treewidth.

One “feature” of algorithmically exploiting tree-cut width is that it requires the solution of a non-trivial dynamic programming step. In previous works, this was carried out mostly by direct translations into Integer Linear Programming instances with few integer variables [11] or by using network flows [12]. In the case of EDP, the dynamic programming step requires us to solve an instance of EDP with a vertex cover of size *k* where every vertex outside of the vertex cover has a degree of 2; we call this problem Simple EDP and solve it in the dedicated Sect. 3. It is worth noting that there is only a very small gap between Simple EDP (for which we provide an XP algorithm in Lemma 4) and graphs with a vertex cover of size 3 (where EDP is known to be NP-hard).

In view of our main result, it is natural to ask whether the algorithm can be improved to a fixed-parameter one. After all, given the parallels between EDP parameterized by tree-cut width (an edge-separator based parameter) and VDP parameterized by treewidth (a vertex-separator based parameter), one would rightfully expect that the fixed-parameter tractability result on the latter [24] would be mirrored in the former case. Surprisingly, we rule this out by showing that EDP parameterized by tree-cut width is W[1]-hard [5, 7] and hence unlikely to be fixed-parameter tractable; in fact, we obtain this lower-bound result even in the more restrictive setting of Simple EDP in Lemma 5. The proof is based on an involved reduction from an adapted variant of the Multidimensional Subset Sum problem [12, 13] and forms our second main contribution.

Having ruled out fixed-parameter algorithms for EDP parameterized by tree-cut width and in view of previous lower-bound results, one may ask whether it is even possible to obtain such an algorithm for any reasonable parameterization. We answer this question positively by using the size of a minimum feedback edge set as a parameter. In fact, we show an even stronger result: as our final contribution, we obtain a so-called *linear kernel* [5, 7] for EDP parameterized by the size of a minimum feedback edge set (Theorem 3).

*Organization of the Paper* After introducing the required preliminaries in Sect. 2, we proceed to introducing Simple EDP, solving it via an XP algorithm and establishing our lower-bound result (Sect. 3). Section 4 then contains our algorithm for EDP parameterized by tree-cut width. Finally, in Sect. 5 we obtain a polynomial kernel for EDP parameterized by the size of a minimum feedback edge set.

## Preliminaries

We use standard terminology for graph theory, see for instance [6]. Given a graph *G*, we let *V*(*G*) denote its vertex set and *E*(*G*) its edge set. The (open) neighborhood of a vertex $x \in V(G)$ is the set $\{y\in V(G):xy\in E(G)\}$ and is denoted by $N_G(x)$. For a vertex subset *X*, the neighborhood of *X* is defined as $\bigcup _{x\in X} N_G(x){\setminus }X$ and denoted by $N_G(X)$; we drop the subscript if the graph is clear from the context. *Contracting* an edge *a*, *b* is the operation of replacing vertices *a*, *b* by a new vertex whose neighborhood is $(N(a)\cup N(b)){\setminus }\{a,b\}$. For a vertex set *A* (or edge set *B*), we use $G-A$ ($G-B$) to denote the graph obtained from *G* by deleting all vertices in *A* (edges in *B*), and we use *G*[*A*] to denote the *subgraph induced on* *A*, i.e., $G- (V(G){\setminus }A)$. A *path segment* of a path *Q* is a path that is a subgraph of *Q*.

A *forest* is a graph without cycles, and an edge set *X* is a *feedback edge set* if $G-X$ is a forest. The *feedback edge set number* of a graph *G*, denoted by ${\mathbf{fes}}(G)$, is the smallest integer *k* such that *G* has a feedback edge set of size *k*. We use [*i*] to denote the set $\{0,1,\ldots ,i\}$.

### Parameterized Complexity

A *parameterized problem* $\mathcal {P}$ is a subset of $\varSigma ^* \times \mathbb {N}$ for some finite alphabet $\varSigma$. Let $L\subseteq \varSigma ^*$ be a classical decision problem for a finite alphabet, and let *p* be a non-negative integer-valued function defined on $\varSigma ^*$. Then *L* *parameterized by* *p* denotes the parameterized problem $\{\,(x,p(x)) \;{|}\;x\in L \,\}$ where $x\in \varSigma ^*$. For a problem instance $(x,k) \in \varSigma ^* \times \mathbb {N}$ we call *x* the main part and *k* the parameter. A parameterized problem $\mathcal {P}$ is *fixed-parameter tractable* (FPT in short) if a given instance (*x*, *k*) can be solved in time $f(k) \cdot |x|^{\mathcal {O}(1)}$ where *f* is an arbitrary computable function of *k*; we call algorithms running in this time *fixed-parameter algorithms*.

Parameterized complexity classes are defined with respect to *fpt-reducibility*. A parameterized problem *P* is *fpt-reducible* to *Q* if in time $f(k)\cdot |x|^{\mathcal {O}(1)}$, one can transform an instance (*x*, *k*) of $\mathcal {P}$ into an instance $(x',k')$ of $\mathcal {Q}$ such that $(x,k)\in \mathcal {P}$ if and only if $(x',k')\in \mathcal {Q}$, and $k'\le g(k)$, where *f* and *g* are computable functions depending only on *k*. Owing to the definition, if $\mathcal {P}$ fpt-reduces to $\mathcal {Q}$ and $\mathcal {Q}$ is fixed-parameter tractable then *P* is fixed-parameter tractable as well. Central to parameterized complexity is the following hierarchy of complexity classes, defined by the closure of canonical problems under fpt-reductions:

$${{{\textsf {FPT}}}}\subseteq {{{{{\textsf {W}}}}}}{{[1]}} \subseteq {{{{{\textsf {W}}}}}}{{[2]}} \subseteq \cdots \subseteq {{{\textsf {XP}}}}.$$

All inclusions are believed to be strict. In particular, ${{{\textsf {FPT}}}}\ne {{{{{\textsf {W}}}}}}{{[1]}}$ under the Exponential Time Hypothesis.

A major goal in parameterized complexity is to distinguish between parameterized problems which are in ${{{\textsf {FPT}}}}$ and those which are ${{{{{\textsf {W}}}}}}{{[1]}}$-hard, i.e., those to which every problem in ${{{{{\textsf {W}}}}}}{{[1]}}$ is fpt-reducible. There are many problems shown to be complete for ${{{{{\textsf {W}}}}}}{{[1]}}$, or equivalently ${{{{{\textsf {W}}}}}}{{[1]}}$-complete, including the Multi-Colored Clique (MCC) problem [7]. We refer the reader to the respective monographs [4, 7, 10] for an in-depth introduction to parameterized complexity.

### Edge-Disjoint Path Problem

Throughout the paper we consider the following problem.

**Table:** 

Edge-Disjoint Paths (EDP)	
Input:	A graph *G* and a set *P* of *terminal pairs*, i.e., a set of subsets of *V*(*G*) of size two.
Question:	Is there a set of pairwise edge-disjoint paths connecting every set of terminal pairs in *P*?

A vertex which occurs in a terminal pair is called a *terminal*, and a set of pairwise edge-disjoint paths connecting every set of terminal pairs in *P* is called a *solution*. Without loss of generality, we assume that *G* is connected. The Vertex-Disjoint Paths (VDP) problem is defined analogously as EDP, with the sole distinction being that the paths must be vertex-disjoint.

The following proposition establishes a link between EDP and VDP on graphs of bounded degree. Since we will not need the notion of *treewidth* [23] for any other result presented in the paper, we refer to the standard textbooks [4, 7] for its definition.

#### Proposition 1

*There exists a linear-time reduction from* EDP *to* VDP *with the following property: if the input graph has treewidth* *k* *and maximum degree* *d*, *then the output graph has treewidth at most* $(k+1)d$.

#### Proof

Let (*G*, *P*) be an instance of EDP where *G* has treewidth *k* and maximum degree *d*; let $V=V(G)$ and $E=E(G)$. Observe that if any vertex $v\in V$ occurs in *P* more than *d* many times, then (*G*, *P*) must be a **NO**-instance (we assume that *P* does not contain tuples in the form (*a*, *a*) for any *a*).

Consider the graph $G'$ obtained in the following two-step procedure. First, we subdivide each edge in *G* (i.e., we replace that edge with a vertex of degree 2 that is adjacent to both endpoints of the original edge); let $V'$ be the set of vertices created by such subdivisions. Second, for each vertex $v=v_1\in V$ of the original graph *G*, we create $d-1$ copies $v_2,\ldots , v_d$ of that vertex and set their neighborhood to match that of $v_1$. This construction gives rise to a natural mapping $\alpha$ from *G* to $G'$ which maps each $v\in V$ to the set $v_1,\ldots ,v_d$ and each $e\in E$ to the vertex created by subdividing *e*. Next, we iteratively process *P* as follows: for each $\{v,w\}\in P$, we add a tuple $\{v', w'\}$ into the set $P'$ such that $v'\in \alpha (v)$, $w'\in \alpha (w)$ and neither $v'$ nor $w'$ occurs in any other pair in $P'$ (the last condition can be ensured because each vertex in *v* has *d* copies in $G'$ but never occurs more than *d* times in *P*).

It is now easy to verify that (*G*, *P*) is a **YES**-instance of EDP if and only if $(G',P')$ is a **YES**-instance of VDP. Indeed, consider a solution *S* (i.e., a set of edge disjoint paths) for (*G*, *P*). For each *v*-*w* path *Q* in *S*, there is a corresponding tuple $(v',w')$ in $P'$, and we can construct a $v'$-$w'$ path $Q'$ by (a) replacing each edge and vertex used by *Q* with a vertex in the $\alpha$-image of that edge and vertex, while (b) ensuring that all paths constructed in this way are pairwise vertex-disjoint. This means that $(G',P')$ is also a **YES**-instance. On the other hand, if $(G',P')$ is a **YES**-instance and this is witnessed by a set $S'$ of vertex-disjoint paths spanning a minimal set of vertices, then by this minimality assumption it follows that each path may only visit the $\alpha$-image of any vertex $v\in V(G)$ at most once. Now consider a path $Q'\in S'$, and notice that $Q'$ can be viewed as a sequence of vertices of the form $(\alpha (v),\alpha (e_1),\alpha (v_1), \alpha (e_2),\ldots ,\alpha (w))$. The sequence obtained from the images of $\alpha$, i.e., $(v,e_1,v_1,e_2,\ldots ,w)$ must then also form a path, and moreover the set of paths obtained in this way must be edge-disjoint by construction.

To conclude the proof, observe that it is possible to convert any tree-decomposition (*T*, *X*) [7] of *G* of width *k* into a tree-decomposition of $G'$ of width $(k+1)d$ by (1) replacing each vertex *v* by $\alpha (v)$ in *T*, and then (2) by choosing, for each edge $e=ab\in E$, a bag $X\supseteq \{a,b\}$, creating a bag $X'=X\cup \{\alpha (e)\}$, and attaching $X'$ to *X* as a leaf.□

We remark that Proposition 1 in combination with the known fixed-parameter algorithm for VDP parameterized by treewidth [24] provides an alternative proof for the fixed-parameter tractability of EDP parameterized by degree and treewidth [13]. Finally, we introduce one bit of useful notation that applies to an instance (*G*, *P*) of EDP: for a subgraph *H* of *G*, we let $P^H_2$ denote the subset of terminal pairs which are subsets of *V*(*H*) and $P^H_1$ denote the subset of terminal pairs with a non-empty intersection with *V*(*H*).

### Tree-Cut Width

The notion of tree-cut decompositions was introduced by Wollan [25], see also [20]. A family of subsets $X_1, \ldots , X_{k}$ of *X* is a *near-partition* of *X* if they are pairwise disjoint and $\bigcup _{i=1}^{k} X_i=X$, allowing the possibility of $X_i=\emptyset$.

#### Definition 1

A *tree-cut decomposition* of *G* is a pair $(T,{\mathcal {X}})$ which consists of a rooted tree *T* and a near-partition ${\mathcal {X}}=\{X_t\subseteq V(G): t\in V(T)\}$ of *V*(*G*). A set in the family ${\mathcal {X}}$ is called a *bag* of the tree-cut decomposition.

For any node *t* of *T* other than the root *r*, let $e(t)=ut$ be the unique edge incident to *t* on the path to *r*. Let $T^u$ and $T^t$ be the two connected components in $T-e(t)$ which contain *u* and *t*, respectively. Note that $(\bigcup _{q\in T^u} X_q, \bigcup _{q\in T^t} X_q)$ is a near-partition of *V*(*G*), and we use $E_t$ to denote the set of edges with one endpoint in each part. We define the *adhesion* of *t* (${\mathbf {adh}}(t)$) as $|E_t|$; we explicitly set ${\mathbf {adh}}(r)=0$ and $E_r=\emptyset$.

The *torso* of a tree-cut decomposition $(T,{\mathcal {X}})$ at a node *t*, written as $H_t$, is the graph obtained from *G* as follows. If *T* consists of a single node *t*, then the torso of $(T,{\mathcal {X}})$ at *t* is *G*. Otherwise let $T_1, \ldots , T_{\ell }$ be the connected components of $T-t$. For each $i=1,\ldots , \ell$, the vertex set $Z_i\subseteq V(G)$ is defined as the set $\bigcup _{b\in V(T_i)}X_b$. The torso $H_t$ at *t* is obtained from *G* by *consolidating* each vertex set $Z_i$ into a single vertex $z_i$ (this is also called *shrinking* in the literature). Here, the operation of consolidating a vertex set *Z* into *z* is to substitute *Z* by *z* in *G*, and for each edge *e* between *Z* and $v\in V(G){\setminus }Z$, adding an edge *zv* in the new graph. We note that this may create parallel edges.

The operation of *suppressing* (also called *dissolving* in the literature) a vertex *v* of degree at most 2 consists of deleting *v*, and when the degree is two, adding an edge between the neighbors of *v*. Given a connected graph *G* and $X\subseteq V(G)$, let the *3-center* of (*G*, *X*) be the unique graph obtained from *G* by exhaustively suppressing vertices in $V(G) {\setminus }X$ of degree at most two. Finally, for a node *t* of *T*, we denote by $\tilde{H}_t$ the 3-center of $(H_t,X_t)$, where $H_t$ is the torso of $(T,{\mathcal {X}})$ at *t*. Let the *torso-size* ${\mathbf {tor}}(t)$ denote $|\tilde{H}_t|$.

#### Definition 2

The width of a tree-cut decomposition $(T,{\mathcal {X}})$ of *G* is $\max _{t\in V(T)}\{ {\mathbf {adh}}(t),$ ${\mathbf {tor}}(t) \}$. The tree-cut width of *G*, or ${\mathbf {tcw}}(G)$ in short, is the minimum width of $(T,{\mathcal {X}})$ over all tree-cut decompositions $(T,{\mathcal {X}})$ of *G*.

**Fig. 1:**
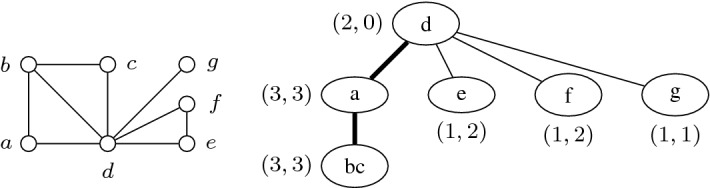
A graph *G* and a width-3 tree-cut decomposition of *G*, including the torso-size (left value) and adhesion (right value) of each node

We also refer to [15] for a novel alternative definition of tree-cut width. Without loss of generality, we shall assume that $X_r=\emptyset$. We conclude this subsection with some notation related to tree-cut decompositions. Given a tree node *t*, let $T_t$ be the subtree of *T* rooted at *t*. Let $Y_t=\bigcup _{b\in V(T_t)} X_b$, and let $G_t$ denote the induced subgraph $G[Y_t]$. A node $t\ne r$ in a rooted tree-cut decomposition is *thin* if ${\mathbf {adh}}(t)\le 2$ and *bold* otherwise (Fig. 1).

While it is not known how to compute optimal tree-cut decompositions efficiently, there exists a fixed-parameter 2-approximation algorithm which we can use instead.

#### Theorem 1

[18] *There exists an algorithm that takes as input an* *n* *-vertex graph* *G* *and integer* *k*, *runs in time* $2^{\mathcal {O}(k^2 \log k)} n^2$, *and either outputs a tree-cut* *decomposition of* *G* *of width at most* 2*k* *or correctly reports that* ${\mathbf {tcw}}(G)> k$.

A tree-cut decomposition $(T,{\mathcal {X}})$ is *nice* if it satisfies the following condition for every thin node $t\in V(T)$: $N_G(Y_t)\cap (\bigcup _{b\,\text{ is\, a \,sibling \,of }\,t}Y_b)=\emptyset$. The intuition behind nice tree-cut decompositions is that we restrict the neighborhood of thin nodes in a way which facilitates dynamic programming.

#### Lemma 1

[11] *There exists a cubic-time algorithm which transforms any rooted tree-cut decomposition* $(T,{\mathcal {X}})$ *of* *G* *into a nice tree-cut decomposition of the same graph, without increasing its width or number of nodes.*

For a node *t* in a nice tree-cut decomposition, we let $B_t=\{\,b\text { is a child of }t$ $\;{|}\;$ ${\mathbf {adh}}(b)\le 2\wedge N_G(Y_b)\subseteq X_t \,\}$ denote the set of thin children of *t* whose neighborhood is a subset of $X_t$, and we let $A_t=\{\,a\text { is a child of }t\;{|}\;a\not \in B_t \,\}$ be the set of all other children of *t*. The following property of nice tree-cut decompositions will be crucial for our algorithm; among others, it implies that only a bounded number of children of *t* contain neighbors of vertices that do not lie in $X_t$.

#### Lemma 2

[11] *Let* *t* *be a node in a nice tree-cut decomposition of width* *k*. *Then* $|A_t|\le 2k+1$.

We refer to previous work [11, 18, 20, 25] for a more detailed comparison of tree-cut width to other parameters. Here, we mention only that tree-cut width lies “between” treewidth and treewidth plus maximum degree.

#### Lemma 3

[11, 20, 25] *Let* ${\mathbf{tw}}(G)$ *denote the treewidth of* *G* *and* ${\mathbf{degtw}}(G)$ *denote the maximum over* ${\mathbf{tw}}(G)$ *and the maximum degree of a vertex in* *G*. *Then* ${\mathbf{tw}}(G)\le 2{\mathbf {tcw}}(G)^2+3{\mathbf {tcw}}(G)$, and ${\mathbf {tcw}}(G)\le 4{\mathbf{degtw}}(G)^2$.

In this context, we can view tree-cut width as a parameter which serves as a “middle ground” for solving EDP. On one hand, EDP remains NP-hard even on graphs of bounded treewidth. On the other hand, parameterizing EDP by ${\mathbf{degtw}}$ yields a fixed-parameter algorithm, but this is only useful on graphs of small maximum degree, where it simply collapses to solving VDP parameterized by treewidth. In this paper, we show that tree-cut width allows for a non-trivial XP (but not a fixed-parameter) algorithm for EDP. We also remark that Lemma 3 immediately implies that VDP is FPT parameterized by tree-cut width.

## The Simple Edge-Disjoint Paths Problem

Before we start working towards our algorithm for solving EDP parameterized by tree-cut width, we will first deal with a simpler (but crucial) setting for the problem. We call this the Simple Edge-Disjoint Paths problem (Simple EDP) and define it below.

**Table:** 

Simple EDP	
Input:	An EDP instance (*G*, *P*) such that $V(G)=A\cup B$ where *B* is an independent set containing vertices of degree at most 2.
Parameter:	$k=|A|$
Question:	Is (*G*, *P*) a **YES**-instance of EDP?

Notice that every instance of Simple EDP has tree-cut width at most *k*, and so it forms a special case of EDP parameterized by tree-cut width. Indeed, the tree-cut decomposition where *T* is a star, the center bag contains *A*, and each leaf bag contains a vertex from *B* (except for the root *r*, where $X_r=\emptyset$), has tree-cut width at most *k*. This contrasts to the setting where *G* has a vertex cover of size 3 and all vertices outside the vertex cover have degree 3; the tree-cut width of such graphs is not bounded by any constant, and EDP is known to be NP-complete in this setting [9].

The main reason we introduce and focus on Simple EDP is that it captures the combinatorial problem that needs to be solved in the dynamic step of the algorithm for EDP parameterized by tree-cut width. Hence, our first task here will be to solve Simple EDP by an algorithm that can later be called as a subroutine.

### Lemma 4

Simple EDP *can be solved in time* $$\mathcal {O}((|P|+1)^{\left( {\begin{array}{c}k\\ 2\end{array}}\right) +1}(k+1)!)$$.

### Proof

We will start by simplifying the instance using some simple observations. First we will show that we can remove all vertices in *B* that are not contained in any terminal pair by adding multi-edges to *G*[*A*]. Namely, let *v* be a vertex in *B* that does not appear in any terminal pair in *P*. If *v* has no neighbors or at most one neighbor, then *v* can simply be removed from *G*, and if *v* has degree two, then we can remove *v* and add an edge between its two neighbors in *A*. Hence in the following we will assume that all vertices in *B* occur in at least one terminal pair and that *G*[*A*] can contain multi-edges.

Let the terminal graph of *G*, denoted $G^T$, be the graph with vertex set *V* and edge set *P*. The following two observations will be crucial for our algorithm:

1. Consider a path *Q* connecting a terminal pair $p \in P$ in a solution. Because *B* is an independent set and every vertex in *B* has degree at most two and is contained in at least one terminal pair in *P*, we obtain that all inner vertices of *Q* are from *A*. Hence, *Q* contains at most $k+2$ vertices and all inner vertices of *Q* are contained in *A*. It follows that *Q* is completely characterized by the sequence of vertices it uses in *A*. Consequently, there are at most $$\sum _{\ell =1}^k\left( {\begin{array}{c}k\\ \ell \end{array}}\right) \ell !\le (k+1)!$$ different types of paths that need to be considered for the connection of any terminal pair.
2. $G^T[B]$ is a disjoint union of paths and cycles. This is because every vertex *v* of *G* can be contained in at most $|N_G(v)|$ terminal pairs in *P* (otherwise we immediately reject) and all vertices in *B* have degree at most two.

Let *u* and *v* be two distinct vertices in *A*. Because $|A|\le k$, we can enumerate all possible paths between *u* and *v* in *G*[*A*] in time $\mathcal {O}((k+1)!)$. We will represent each such path *H* as a binary vector $E^H$, whose entries are indexed by all sets of two distinct vertices in *A*, such that $E^H[e]=1$ if *H* uses the edge *e* and $E^H[e]=0$ otherwise. Moreover, we will denote by $E_{u,v}$ the set $\{\,E^H \;{|}\;H\, is\, \,a \,path \,between \,u \,and \,v \,in \,G[A]\,\}$; intuitively, $E_{u,v}$ captures all possible sets of edges that can be used in order to connect *u* to *v*.

Let *S* be a solution for (*G*, *P*). The algorithm represents every solution *S* for (*G*, *P*) as a solution vector $E^S$ of natural numbers whose entries are indexed by all sets $\{u,v\}$ of two distinct vertices in *A*. More specifically, for two distinct vertices *u* and *v* in *A*, $E^S[\{u,v\}]$ is equal to the number of edges between *u* and *v* used by the paths in *S*. The algorithm uses dynamic programming to compute the set $\mathcal {L}$ of all solution vectors; clearly, $\mathcal {L}\ne \emptyset$ if and only if (*G*, *P*) is a **YES**-instance. We compute $\mathcal {L}$ in two main steps:

1. the algorithm computes the set $\mathcal {L}_A$ of all solution vectors for the sub-instance $(G[A],P')$ of (*G*, *P*), where $P'$ is the subset of *P* containing all terminal pairs $\{p,q\}$ with $p,q \in A$.
2. the algorithm computes the set of all solution vectors for the sub-instance $(G,P{\setminus }P')$. Note that every terminal pair *p* in $P{\setminus }P'$ is either completely contained in *B*, in which case it forms an edge of a path or a cycle in $G^T[B]$, or *p* has one vertex in *A* and the other vertex in *B*, which is the endpoint of a path in $G^T[B]$. The algorithm now computes the set of all solution vectors for the sub-instance $(G,P{\setminus }P')$ in two steps:
  (S2A) For every cycle *C* in $G^T[B]$, the algorithm computes the set $\mathcal {L}_C$ of all solution vectors for the sub-instance $(G[A \cup V(C)], P^C_2)$, where $P^C_2$ is the set of all terminal pairs in *P* with both terminals in *C*.
  (S2B) For every path *H* in $G^T[B]$, the algorithm computes the set $\mathcal {L}_H$ of all solution vectors for the sub-instance $(G[A\cup V(H)],P^H_1)$, where $P^H_1$ is the set of all terminal pairs in *P* with at least one endpoint in *H*.

In the end, the set of all *hypothetical solution vectors* $\mathcal {L}'$ for (*G*, *P*) is obtained as $\mathcal {L}_A\oplus (\oplus _{C is a cycle of G^T[B]}\mathcal {L}_C)\oplus (\oplus _{H is a path of G^T[B]}\mathcal {L}_H)$, where $\mathcal {P}\oplus \mathcal {P}'$ for two sets $\mathcal {P}$ and $\mathcal {P}'$ of solution vectors is equal to $\{\,R+R' \;{|}\;R \in \mathcal {P}\wedge R'\in \mathcal {P}'\,\}$. Each vector in $\mathcal {L}'$ describes one possible set of multi-edges in *G*[*A*] that can be used to connect all terminal pairs in *P*. In order to compute $\mathcal {L}$, one simply needs to remove all vectors from $\mathcal {L}'$ which require more multi-edges than are available in *G*[*A*]; in particular, to obtain $\mathcal {L}$ we delete each vector $E^S$ from $\mathcal {L}'$ such that there exist $u,v\in A$ where $E^S[\{u,v\}]$ exceeds the number of multi-edges between *u* and *v* in *G*. The algorithm then returns **YES** if $\mathcal {L}$ is non-empty and otherwise the algorithm returns **NO**. Note that, as is usually the case with these types of dynamic programming algorithms, the algorithm can also be easily modified to find a solution for (*G*, *P*), without increasing its running time.

The set $\mathcal {L}_A$ described in step (S1) is computed as follows. Given an arbitrary but fixed ordering $p_1,\ldots ,p_{|P'|}$ of the terminal pairs in $P'$, let $P_i$ be the set $\{\,p_j \;{|}\;1\le j \le i\,\}$, for every *i* with $1 \le i \le |P'|$. The algorithm now uses dynamic programming to compute the sets $S_1,\ldots ,S_{|P'|}$, where $S_i$ contains the set of all hypothetical solution vectors for the instance $(G[A],P_i)$ as follows. The algorithm starts by setting $T_1$ to be the set $E_{p_1}$. Then for every *i* with $1< i\le |P'|$, the algorithm computes $T_i$ from $T_{i-1}$ as the set $\{\,E+E' \;{|}\;E \in T_{i-1}\wedge E' \in E_{p_i}\,\},$

The set $\mathcal {L}_C$ described in step (S2A) for a cycle $C=(v_1,\ldots ,v_n)$ of $G^T[B]$ is computed as follows. Note that every vertex in *C* has exactly two neighbors in *A* (and also in *G*). For a neighbor *n* of $v_i$, we denote by $\bar{n}$ the other neighbor of $v_i$ in *G*, i.e., $\bar{n}$ is the unique neighbor in $N_G(v_i){\setminus }\{n\}$. For every *i* with $2 \le i \le n$, we denote by $P_i$ the set $\{\,\{v_j,v_{j+1}\}\;{|}\;1 \le j < i\,\}$ of terminal pairs. The algorithm starts by computing a table $T_i$ for every *i* with $2\le i \le n$. Informally, for every neighbor $n_1$ of $v_1$ and every neighbor $n_i$ of $v_i$ in *G*, the table $T_i$ contains all hypothetical solution vectors for the instance induced on *A* and the vertices $v_1,\ldots ,v_i$ that use $n_1$ to connect the terminal pair $\{v_1,v_2\}$ and $n_i$ to connect the terminal pair $\{v_{i-1},v_i\}$. More formally, for every $n_1 \in N_G(v_1)$ and $n_i \in N_G(v_i)$ the table $T_i$ contains the set of all solution vectors for the instance $(G[A\cup \{v_1,\ldots ,v_i\}]-\{v_1\bar{n}_1,v_i\bar{n}_i\},P_i)$.

The tables $T_2,\ldots ,T_n$ are iteratively computed starting with $T_2$ as follows. For every $n_1 \in N_G(v_1)$ and $n_2 \in N_G(v_2)$, $T_2[n_1,n_2]$ is equal to $E_{n_1,n_2}$. Moreover, for every *i* with $3\le i \le n$, the table $T_i$ is obtained from the table $T_{i-1}$ as follows. For every $n_1 \in N_G(v_1)$ and $n_i \in N_G(v_i)$, $T_i[n_1,n_i]$ is equal to the union of the following two sets:

- $$\{\,E+E' \;{|}\;E \in T_{i-1}[n_1,n_{i-1}] \wedge E' \in E_{\bar{n}_{i-1},n_i} \,\}$$ and
- $$\{\,E+E' \;{|}\;E \in T_{i-1}[n_1,\bar{n}_{i-1}] \wedge E' \in E_{n_{i-1},n_i} \,\}$$

where $\{n_{i-1},\bar{n}_{i-1}\}=N_G(v_{i-1})$. Finally, the set of all hypothetical solution vectors for the instance $(G[A\cup C],P^C_2)$ is obtained from the table $T_n$ as the union of the sets $\{\,E+E' \;{|}\;E \in T_n[n_1,n_n] \wedge E' \in E_{\bar{n}_n,\bar{n}_1}\,\}$ for every $n_1 \in N_G(v_1)$ and every $n_n\in N_G(v_n).$

The set $\mathcal {L}_H$ described in step (S2B) for a path $H=(v_1,\ldots ,v_n)$ of $G^T[B]$ is computed as follows. Note first that every inner vertex of *H* has exactly two neighbors in *A* and the two endpoints $v_1$ and $v_n$ of *H* have either one or two neighbors in *A*. We will compute $\mathcal {L}_H$ with the help of the table $T_n$ computed for the step (S2A) above. First note that if both endpoints $v_1$ and $v_n$ of *H* have only one neighbor in *A*, then $\mathcal {L}_H$ is equal to $T_n[n_1,n_n]$, where $n_1$ and $n_n$ are the unique neighbors of $v_1$ and $v_n$, respectively, in *G*. Moreover, if both endpoints occur only in one terminal pair (but could have up to two neighbors in *G*), then $\mathcal {L}_H$ is equal to the union of the sets $T_n[n_1,n_n]$ for every neighbor $n_1 \in N_G(v_1)$ and every neighbor $n_n\in N_G(v_n)$. Now consider the case that both endpoints $v_1$ and $v_n$ occur in exactly two terminal pairs; the case that only one of them occurs in two terminal pairs is then analogously. Then $v_1$ occurs in the terminal pair $\{v_1,v_2\}$ and in the terminal pair $\{v_1,a_1\}$ for some $a_1 \in A$ and similarily $v_n$ occurs in the terminal pair $\{v_{n-1},v_n\}$ and in the terminal pair $\{v_n,a_n\}$ for some $a_n \in A$. In this case, $\mathcal {L}_H$ is equal to the union of the sets $\{\,E+E'+E'' \;{|}\;E \in E_{\bar{n}_1,a_1} \wedge E' \in T_n[n_1,n_n] \wedge E'' \in E_{\bar{n}_n,a_n}\,\}$ for every $n_1 \in N_G(v_1)$ and every $n_n\in N_G(v_n)$. All other remaining cases can be handled analogously.

This completes the description of the algorithm. To verify correctness, one can observe that each solution vector computed by the algorithm can be traced back to a specific choice of edges (a path) that connects each terminal pair in *P*, and since there are sufficient multi-edges in *G*[*A*] to accommodate all the resulting paths, this guarantees the existence of a solution. On the other hand, if a solution exists then it surely has a solution vector, and moreover the algorithm will discover this solution vector by choosing, for each $\{a,b\} \in P$, the entry in $E^H$ which corresponds to the *a*-*b* path used in the solution.

Finally, we establish the running time bound. Note first that every set of solution vectors computed at any point in the algorithm contains at most $$(|P|+1)^{\left( {\begin{array}{c}k\\ 2\end{array}}\right) }$$ elements. Moreover, as argued in (O1) the set $E_{u,v}$ for two distinct vertices *u* and *v* in *A* can be computed in time $\mathcal {O}((k+1)!)$ and contains at most $(k+1)!$ elements. From this it follows that the time required to compute $\mathcal {L}_A$ in (S1) is at most $$\mathcal {O}((|P|+1)^{\left( {\begin{array}{c}k\\ 2\end{array}}\right) }(k+1)!|P'|)$$. Similarly, the time required to compute $\mathcal {L}_C$ for a cycle *C* in $G^T[B]$ in step (S2A) is at most $$\mathcal {O}((|P|+1)^{\left( {\begin{array}{c}k\\ 2\end{array}}\right) }(k+1)!|P^C_2|)$$ and the time required to compute $\mathcal {L}_H$ for a path *H* in $G^T[B]$ in step (S2B) is at most $$\mathcal {O}((|P|+1)^{\left( {\begin{array}{c}k\\ 2\end{array}}\right) }(k+1)!|P^H_1|)$$. Hence the time required to compute $\mathcal {L}_A$ together with all the sets $\mathcal {L}_C$ and $\mathcal {L}_H$ for every cycle *C* and path *H* of $G^T[B]$ is at most $$\mathcal {O}((|P|+1)^{\left( {\begin{array}{c}k\\ 2\end{array}}\right) }(k+1)!|P|)$$. Finally, combining these sets into $\mathcal {L}'$ does not incur an additional run-time overhead since $\mathcal {L}'$ can be computed iteratively as part of the computation of the sets $\mathcal {L}_A$, $\mathcal {L}_C$, and $\mathcal {L}_H$.□

Notice that Lemma 4 does not provide a fixed-parameter algorithm for Simple EDP. Our second task for this section will be to rule out the existence of such algorithms (hence also ruling out the fixed-parameter tractability of EDP parameterized by tree-cut width).

Before we proceed, we would like note that this outcome was highly surprising for the authors. Indeed, not only does this “break” the parallel between {VDP, treewidth} and {EDP, tree-cut width}, but inspecting the dynamic programming algorithm for EDP parameterized by tree-cut width presented in Sect. 4 reveals that solving Simple EDP is the only step which requires more than “FPT-time”. In particular, if Simple EDP were FPT, then EDP parameterized by tree-cut width would also be FPT. This situation contrasts the vast majority of dynamic programming algorithms for parameters such as treewidth and clique-width [3], where the complexity bottleneck is usually tied to the size of the records used and not to the computation of the dynamic step.

Our lower-bound result is based on a fpt-reduction from the following problem:

**Table:** 

Multidimensional Subset Sum (MSS)	
Input:	An integer *k*, a set $S=\{s_1,\ldots ,s_n\}$ of item-vectors with $s_i \in \mathbb {N}^{k}$ for every *i* with $1\le i \le n$, a target vector $t \in \mathbb {N}^k$, and an integer $\ell$.
Parameter:	*k*
Question:	Is there a subset $S' \subseteq S$ with $|S'|\ge \ell$ such that $\sum _{s \in S'}s\le t$?

The W[1]-hardness of MSS can be obtained by a trivial reduction from the following problem, which was recently shown to be W[1]-hard by Ganian, Ordyniak and Ramanujan [13]:

**Table:** 

Multidimensional Relaxed Subset Sum (MRSS)	
Input:	An integer *k*, a set $S=\{s_1,\ldots ,s_n\}$ of item-vectors with $s_i \in \mathbb {N}^{k}$ for every *i* with $1\le i \le n$, a target vector $t \in \mathbb {N}^k$, and an integer $\ell$.
Parameter:	*k*
Question:	Is there a subset $S' \subseteq S$ with $|S'|\le \ell$ such that $\sum _{s \in S'}s\ge t$?

Indeed, given an instance $(k,S,t,\ell )$ of MRSS, it is straightforward to verify that (*k*, *S*,  $(\sum _{s \in S}s)-t,|S|-\ell )$ is an equivalent instance of MSS; since the reduction preserves the parameter, this shows that MSS is also W[1]-hard.

### Lemma 5

Simple EDP *is* W[1]-*hard.*

### Proof

We provide a fpt-reduction from MSS. Namely, given an instance $(k,S,t,\ell )$ of MSS, we will construct an equivalent instance (*G*, *P*) with partition *A* and *B* and $|A|=k+3$ of Simple EDP. For convenience and w.l.o.g. we will assume that all entries of the vectors in *S* as well as all entries of the target vector *t* are divisible by two; furthermore, we will describe the constructed instance of Simple EDP with multi-edges between vertices in *A* (note that these can be replaced by degree-2 vertices in *B*, similarly as in Lemma 4).

The graph *G*[*A*] has vertices *a*, *b*, *d*, and $d_1,\ldots ,d_k$ and the following multi-edges:

- $|S|-\ell$ edges between *a* and *b*,
- for every *i* with $1 \le i \le k$, *t*[*i*] edges between *d* and $d_i$.

Moreover, for every $s \in S$ we construct a gadget *G*(*s*) consisting of:

- the vertices $v^s,v^s_1,u^s_1,\ldots ,v^s_{\bar{s}},u^s_{\bar{s}}$ with $\bar{s}=\sum _{i=1}^k s[i]$,
- two edges $v^sa$ and $v^sd$,
- for every *i* with $1 \le i \le \bar{s}$, two edges $v_i^sb$ and $u_i^sb$,
- for every *i* with $1\le i \le \bar{s}$ and *i* even, two edges $v^s_id$ and $u^s_id$,
- for every *j* with $1 \le j \le k$ and every *i* with $\sum _{l=1}^{j-1}s[l] < i \le \sum _{l=1}^{j}s[l]$ and *i* odd, two edges $v^s_id_j$ and $u^s_id_j$,
- the terminal pair $\{v^s,v^s_1\}$,
- for every *i* with $1 \le i \le \bar{s}$, a terminal pair $\{v^s_i,u^s_i\}$,
- for every *i* with $1 \le i < \bar{s}$, a terminal pair $\{u^s_i,v^s_{i+1}\}$,

**Fig. 2:**
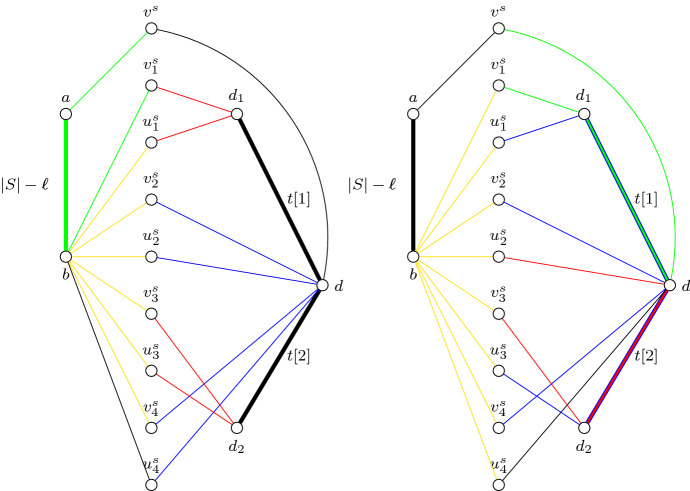
An illustration of the graph *G*[*A*] together with the gadget *G*(*s*) for $k=2$, $s[1]=2$, and $s[2]=2$. Bold edges indicate multi-edges with multiplicities given as an edge label. The left side illustrates configuration (C1) and the right side illustrates configuration (C2) as defined in Claim 1; here the non-black edges indicate the edges used by a solution that uses the corresponding configuration to connect the terminal pairs of *G*(*s*). In particular, on the left side illustrating the case (C1), we have that: the green edges connect the terminal pair $\{v^s,v^s_1\}$, the yellow edges connect the terminal pairs $\{u_i^s,v_{i+1}^s\}$, the blue edges connect the terminal pairs $\{v_i^s,u_i^s\}$ for *i* even, and the red edges connect the terminal pairs $\{v_i^s,u_i^s\}$ for *i* odd. Moreover, on the right side illustrating the case (C2), we have that: the green edges connect the terminal pair $\{v^s,v^s_1\}$, the yellow edges connect the terminal pairs $\{u_i^s,v_{i}^s\}$, the blue edges connect the terminal pairs $\{u_i^s,v_{i+1}^s\}$ for *i* odd, and the red edges connect the terminal pairs $\{u_i^s,v_{i+1}^s\}$ for *i* even (Color figure online)

Then *G* consists of the graph *G*[*A*] together with the vertices and edges of the gadget *G*(*s*) for every $s \in S$; note that *B* is the union of *V*(*G*(*s*)) over every $s \in S$. Moreover, *P* consists of all terminal pairs of the gadgets *G*(*s*) for every $s \in S$. This completes the construction of the instance (*G*, *P*); an illustration is provided in Fig. 2. It remains to show that the instance $(k,S,t,\ell )$ of MSS has a solution if and only if so does the instance (*G*, *P*) of EDP.

We start by showing that there are only two ways to connect all terminal pairs of the gadget *G*(*s*) for every $s \in S$. Figure 2 illustrates the edges used by the two configurations.

### Claim 1

*Let* $\mathcal {S}$ *be a solution for* (*G*, *P*), *and* $s \in S$. *Then either:*

1. *The terminal pair* $\{v^s,v^s_1\}$ *is connected by the path* $(v^s,a,b,v^s_1)$ *and:*
  *for every* *i* *with* $1\le i < \bar{s}$, *the terminal pair* $\{u^s_i,v^s_{i+1}\}$ *is connected by the path* $(u^s_i,b,v^s_{i+1})$,
  *for every i with* $1\le i \le \bar{s}$ *and* *i* *even, the terminal pair* $\{v^s_i,u^s_i\}$ *is connected by the path* $(v^s_i,d,u^s_i)$, *and*
  *for every* *i* *with* $1\le i \le \bar{s}$ *and* *i* *odd, the terminal pair* $\{v^s_i,u^s_i\}$ *is connected by the path* $(v^s_i,d_j,u^s_i)$, *where* *j* *is such that* $\sum _{l=1}^{j-1}s[l] <i\le \sum _{l=1}^{j}s[l]$.
2. *The terminal pair* $\{v^s,v^s_1\}$ *is connected by the path* $(v^s,d,d_j,v^s_1)$, *where* *j* *is the minimum integer such that* $s[j]\ne 0$ *and:*
  *for every* *i* *with* $1\le i \le \bar{s}$, *the terminal pair* $\{v^s_i,u^s_{i}\}$ *is connected by the path* $(v^s_i,b,u^s_{i})$,
  *for every* *i* *with* $1\le i < \bar{s}$ *and* *i* *is odd, the terminal pair* $\{u^s_i,v^s_{i+1}\}$ *is connected by the path* $(u^s_i,d_j,d,v^s_{i+1})$, *where* *j* *is such that* $\sum _{l=1}^{j-1}s[l] < i \le \sum _{l=1}^js[l]$,
  *for every* *i* *with* $1\le i < \bar{s}$ *and* *i* *is even, the terminal pair* $\{u^s_i,v^s_{i+1}\}$ *is connected by the path* $(u^s_i,d,d_j,v^s_{i+1})$, *where* *j* *is such that* $\sum _{l=1}^{j-1}s[l] < i \le \sum _{l=1}^js[l]$.

### Proof

Let $\mathcal {S}$ be a solution for (*G*, *P*) and $s \in G(s)$. Then $\mathcal {S}$ has to connect the terminal pair $\{v^s,v^s_1\}$ either by the path $(v^s,a,b,v^s_1)$ or by the path $(v^s,d,d_j,v^s_1)$.

In the former case, the only way to connect the terminal pair $\{v^s_1,u^s_1\}$ is the path $(v^s_1,d_j,u^s_1)$, where *j* is such that $\sum _{l=1}^{j-1}s[l] <1\le \sum _{l=1}^{j}s[l]$. But then the terminal pair $\{u^s_1,v^s_2\}$ can only be connected by the path $(u^s_1,b,v^s_2)$ and in turn the terminal pair $\{v^s_2,u^s_2\}$ can only be connected by the path $(v^s_2,d,u^s_2)$. Since this pattern continues in this manner, this concludes the argument for the first case.

In the later case, the only way to connect the terminal pair $\{v^s_1,u^s_1\}$ is the path $(v^s_1,b,u^s_1)$. But then the terminal pair $\{u^s_1,v^s_2\}$ can only be connected by the path $(u^s_1,d_j,d,v^s_2)$, where *j* is such that $\sum _{l=1}^{j-1}s[l] <1\le \sum _{l=1}^{j}s[l]$, and in turn the terminal pair $\{v^s_2,u^s_2\}$ can only be connected by the path $(v^s_2,b,u^s_2)$. Finally, the terminal pair $\{u^s_2,v^s_3\}$ can then only be connected by the path $(u^s_2,d,d_j,v^s_3)$, where *j* is such that $\sum _{l=1}^{j-1}s[l] <1\le \sum _{l=1}^{j}s[l]$. Since this pattern continues in this manner, this concludes the argument for the second case.□

Let $\mathcal {S}$ be a solution for (*G*, *P*) and $s \in S$. It follows from Claim 1 that if $\mathcal {S}$ connects the terminal pairs of *G*(*s*) according to (C1), then the only edge used from *G*[*A*] is the edge *ab*. On the other hand, if $\mathcal {S}$ connects the terminal pairs in *G*(*s*) according to (C2), then $\mathcal {S}$ uses *s*[*i*] edges between *d* and $d_j$ for every *i* with $1\le i \le k$.

Towards showing the forward direction, let $S' \subseteq S$ be a solution for $(k,S,t,\ell )$. W.l.o.g. we can assume that $|S'|=\ell$. We claim that the set of edge-disjoint paths $\mathcal {S}$, which if $s \in S'$ connects all terminal pairs in *G*(*s*) according to (C2) and if $s \in S{\setminus }S'$ connects all terminal pairs in *G*(*s*) according to (C1) is a solution for (*G*, *P*). This holds because there are $|S|-\ell$ edges between *a* and *b*, which are sufficient for the elements in $S{\setminus }S'$ to be connected according to (C1). Moreover, because $\sum _{s\in S'}s\le t$, the *t*[*i*] edges between *d* and $d_i$ for every *i* with $1 \le i \le k$, suffice for the elements in $S'$ to be connected according to (C2).

For the reverse direction, let $\mathcal {S}$ be a solution for (*G*, *P*).

We claim that the subset $S'$ of *S* containing all $s \in S$ such that $\mathcal {S}$ connects all terminal pairs in *G*(*s*) according to C2 is a solution for $(k,S,t,\ell )$. Because there are at most $|S|-\ell$ edges between *a* and *b* in *G*[*A*], we obtain that $|S'|\ge \ell$. Moreover, because there are at most *t*[*i*] edges between *d* and $d_i$ in *G*[*A*], it follows that $\sum _{s\in S'}s\le t$. Consequently, $S'$ is a solution for $(k,S,t,\ell )$.□

## An Algorithm for EDP on Graphs of Bounded Tree-Cut Width

The goal of this section is to provide an XP algorithm for EDP parameterized by tree-cut-width. The core of the algorithm is a dynamic programming procedure which runs on a nice tree-cut decomposition $(T,{\mathcal {X}})$ of the input graph *G*.

### Overview

Our first aim is to define the data table the algorithm is going to dynamically compute for individual nodes of the tree-cut decomposition; to this end, we introduce two additional notions. For a node *t*, we say that $Y_t$ (or $G_t$) contains an *unmatched* terminal *s* if $\{s,e\}\in P$, $s\in Y_t$ and $e\not \in Y_t$; let $U_t$ be the multiset containing all unmatched terminals in $Y_t$ (one entry in $U_t$ per tuple in *P* which contains an unmatched terminal). For a subgraph *H* of *G*, let $P^H_2\subseteq P$ denote the subset of terminal pairs whose both endpoints lie in *H*.

Let a *record* for node *t* be a tuple $(\delta , I, F, L)$ where:

- $\delta$ is a partitioning of $E_t$ into four subsets: an even-sized set $I'$ (*internal*), a set $L'$ (*leaving*), an even-sized set $F'$ (*foreign*) and a set $(U')$ (*unused*);
- *I* is a set of subsets of size 2 of $I'$ that is a perfect matching between the edges in $I'$;
- *F* is a set of subsets of size 2 of $F'$ that is a perfect matching between the edges in $F'$;
- *L* is a perfect matching between $U_t$ and the edges in $L'$.

Intuitively, a record captures all the information we need about one possible interaction between a solution to EDP and the edges in $E_t$. In particular, unmatched terminals need to cross between $Y_t$ and $G-Y_t$ using an edge in $E_t$ and *L* captures the first edge used by a path from an unmatched terminal in the solution while $L'$ is the set of all edges in $E_t$ that are used for this purpose. *I* and *F* then capture information about paths which intersect with $E_t$ but whose terminals both lie in $Y_t$ and $G- Y_t$, respectively,^1^ and the sets $I'$ and $F'$ contain all edges used for these two purposes. Finally, the set $U'$ simply contains edges which are not used by a given solution. We formalize this intuitive description below through the notion of a *valid record*.

**Fig. 3:**
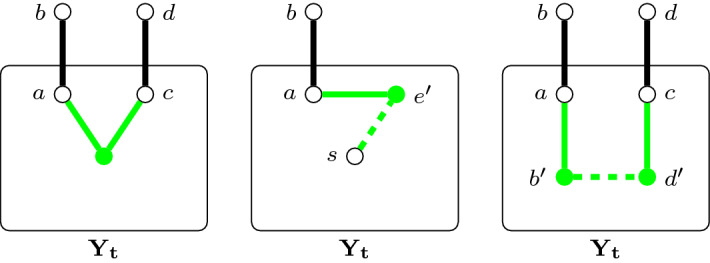
Illustration of the construction of $(G^{t,\lambda },P^{t,\lambda })$ from $(G_t, P^{G_t}_2)$ and $\lambda$. Green vertices and edges represent new elements that are added to $(G^{t,\lambda },P^{t,\lambda })$ and dashed edges represent terminal-pairs. The left, middle, and right picture corresponds to the steps 2, 3, and 4 in the algorithm for constructing $(G^{t,\lambda },P^{t,\lambda })$, respectively (Color figure online)

Let $\lambda =(\delta , I, F, L)$ be a record for *t*. Then, the instance $(G^{t,\lambda },P^{t,\lambda })$ is obtained from $(G_t, P^{G_t}_2)$ and $\lambda$ by the following algorithm (see Fig. 3 for an illustration):

1. initialize $G^{t,\lambda }$ to $G_t$ and $P^{t,\lambda }$ to $P^{G_t}_2$,
2. For each $\{\{a,b\},\{c,d\}\}\in I$ where $a,c\in Y_t$, add a new vertex into $G^{t,\lambda }$ and connect it to *a* and *c* by edges (note that if $a=c$ then this simply creates a new leaf and hence this operation can be ignored).
3. For each $\{s,\{a,b\}\}\in L$ where $a\in Y_t$, add a new tuple $\{s,e'\}$ into $P^{t,\lambda }$ and a new leaf $e'$ into $G^{t,\lambda }$ adjacent to *a*.
4. For each $\{\{a,b\},\{c,d\}\}\in F$ where $a,c\in Y_t$, add two new leaves $b', d'$ into $G^{t,\lambda }$, make them adjacent to *a* and *c* respectively, and add $\{b',d'\}$ into $P^{t,\lambda }$.

#### Definition 3

A record $\lambda =(\delta , I, F, L)$ is *valid* for *t* if $(G^{t,\lambda },P^{t,\lambda })$ is a **YES**-instance of EDP.

We are now ready to define our data tables: for a node $t\in V(T)$, let *D*(*t*) be the set of all valid records for *t*. We now make two observations. First, for any node *t* in a nice tree-cut decomposition of width *k*, it holds that there exist at most $4^k\cdot k!$ distinct records and hence $|D(t)|\le 4^k\cdot k!$; indeed, there are $4^k$ possible choices for $\delta$, and for each such choice and each edge *e* in $E_t$ one has at most *k* options of what to match with *e*. Second, if *r* is the root of *T*, then either $D(r)=\emptyset$ or $D(r)=\{(\emptyset , \emptyset , \emptyset , \emptyset )\}$; furthermore, (*G*, *P*) is a **YES**-instance if and only if the latter holds. Hence it suffices to compute *D*(*r*) in order to solve EDP.

The next lemma shows that *D*(*t*) can be computed efficiently for all leaves of *t*.

#### Lemma 6

*Given* (*G*, *P*), *a width*-*k* *tree-cut decomposition* $(T,{\mathcal {X}})$ *of* *G* *and a leaf* $t\in V(T)$ *as the input, it is possible to compute* *D*(*t*) *in time* $k^{\mathcal {O}(k^2)}$.

#### Proof

We proceed as follows. For each record $\lambda$ for *t*, we construct the instance $(G^{t,\lambda },P^{t,\lambda })$ as per Definition 3 and check whether $(G^{t,\lambda },P^{t,\lambda })$ is a **YES**-instance of EDP. Since $V(G^{t,\lambda })\le 2k$, a simple brute-force algorithm will suffice here. For instance, one can enumerate all partitions of the at most $4k^2$ edges in $G^{t,\lambda }$, and for each such partition one can check whether this represents a set of edge-disjoint paths which forms a solution to $(G^{t,\lambda },P^{t,\lambda })$. If $(G^{t,\lambda },P^{t,\lambda })$ is a **YES**-instance of EDP then we add $\lambda$ into *D*(*t*), and otherwise we do not.

The number of partitions of a set of size $4k^2$ is upper-bounded by $k^{\mathcal {O}(k^2)}$ [1], and $|D(t)|\le 4^k\cdot k!$. Hence the runtime of the whole algorithm described above is dominated by $k^{\mathcal {O}(k^2)}$.□

At this point, all that is left to obtain a dynamic leaves-to-root algorithm which solves EDP is the dynamic step, i.e., computing the data table for a node $t\in V(t)$ from the data tables of its children. Unfortunately, that is where all the difficulty of the problem lies, and our first step towards handling this task will be the introduction of two additional notions related to records. The first is *correspondence*, which allows us to associate each solution to (*G*, *P*) with a specific record for *t*; on an intuitive level, a solution corresponds to a particular record if that record precisely captures the “behavior” of that solution on $E_t$. Correspondence will, among others, later be used to establish the correctness of our algorithm.

#### Definition 4

A solution *S* to (*G*, *P*) *corresponds* to a record $\lambda =(\delta , I, F, L)$ for *t* if the conditions **1.**-**4.** stated below hold for every *a*-*b* path $Q\in S$ such that $Q\cap E_t\ne \emptyset$. We let $s=|Q\cap E_t|$ and we denote individual edges in $Q\cap E_t$ by $e_1,e_2,\ldots e_{s}$, ordered from the edge nearest to *a* along *Q*.

1. If $a,b\not \in Y_t$, then for each odd $i\in [s]$, *F* contains $\{e_i,e_{i+1}\}$.
2. If $a,b \in Y_t$, then for each odd $i\in [s]$, *I* contains $\{e_i,e_{i+1}\}$.
3. If $\{a,b\} \cap Y_t=\{a\}$, then *L* contains $(a,e_1)$, and for each even $i\in [s]$ *F* contains $\{e_{i},e_{i+1}\}$.
4. There are no elements in *I*, *F*, *L* other than those specified above.

Note that “restricting” the solution *S* to the instance $(G^{t,\lambda }, P^{t,\lambda })$ used in Definition 3 yields also a solution to $(G^{t,\lambda }, P^{t,\lambda })$; in particular, for each path $Q\in S$ that intersects $E_t$, one replaces the path segments of *Q* in $G{\setminus }Y_t$ by the newly created vertices to obtain a solution to $(G^{t,\lambda }, P^{t,\lambda })$. Consequently, if *S* corresponds to $\lambda$ then $\lambda$ must be valid (however, it is clearly not true that every valid record has a solution to the whole instance that corresponds to it). Moreover, since Definition 4 is constructive and deterministic, for each solution *S* and node *t* there exists precisely one corresponding valid record $\lambda$.

The second notion that we will need is that of *simplification*. This is an operation which takes a valid record $\lambda$ for a node *t* and replaces $G_t$ by a “small representative” so that the resulting graph retains the existence of a solution corresponding to $\lambda$. Simplification can also be seen as being complementary to the construction of $(G^{t,\lambda },P^{t,\lambda })$ used in Definition 3 (instead of modeling the implications of a record on $G_t$, we model its implications on $G-Y_t$), and will later form an integral part of our procedure for computing valid records for nodes.

**Fig. 4:**
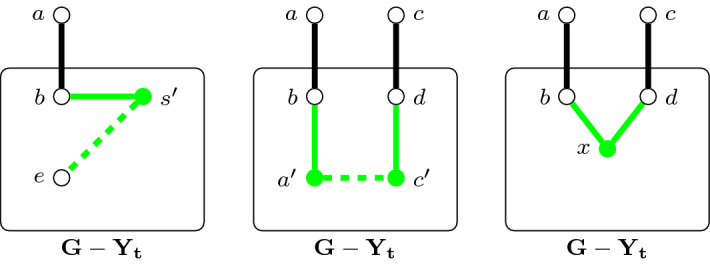
Illustration of the simplification of *t* in accordance with $\lambda$. Green vertices and edges represent new elements that are added to $(G',P')$ and dashed edges represent terminal-pairs. The left, middle, and right picture corresponds to the steps 2, 3, and 4 in the algorithm given in Definition 5, respectively (Color figure online)

#### Definition 5

The *simplification* of a node *t* in accordance with $\lambda =(\delta , I, F, L)$ is an operation which transforms the instance (*G*, *P*) into a new instance $(G',P')$ obtained from $(G-Y_t,P^{G-Y_t}_2)$ and $\lambda$ by the following algorithm (see Fig. 4 for an illustration):

1. initialize $G'$ to $G-Y_t$ and $P'$ to $P^{G-Y_t}_2$,
2. For each $\{s,\{a,b\}\}\in L$ where $(s,e)\in P$ and $b\not \in Y_t$, add a new vertex $s'$ adjacent to *b* to $G'$ and add $(s',e)$ to $P'$.
3. For each $\{\{a,b\},\{c,d\}\}\in I$ where $a,c\in Y_t$ and $a\ne c$, add vertices $a'$ and $c'$ into $G'$ and make them adjacent to *b* and *d* respectively, and add $(a',c')$ into $P'$.
4. For each $\{\{a,b\},\{c,d\}\}\in F$ where $a,c\in Y_t$ and $b\ne d$, add a new vertex *x* to $G'$ and make it adjacent to *b* and *d*.

With regards to simplification, observe that every vertex added to $G-Y_t$ has degree at most 2 and that simplification can never increase the degree of vertices in $G-Y_t$.

#### Observation 1

*If there exists a solution to* (*G*, *P*) *which* *corresponds* *to a record* $\lambda =(\delta , I, F, L)$ *for* *t*, *and if* $(G',P')$ *is the result of simplification of* *t* *in accordance with* $\lambda$, *then* $(G',P')$ *admits a solution. On the other hand, if* $(G',P')$ *is the result of simplification of* *t* *in accordance with a valid record* $\lambda$ *and if* $(G',P')$ *admits a solution, then* (*G*, *P*) *also admits a solution.*

#### Proof

For the forward direction, consider a solution *S* to (*G*, *P*) which corresponds to $\lambda =(\delta , I, F, L)$. Comparing Definition 4 with Definition 5, we observe the following:

1. for each *s*-*e* path $Q\in S$ such that $s,e\not \in Y_t$ and $Q\cap E_t\ne \emptyset$, it holds that each path segment of *Q* in $Y_t$ begins and ends with a pair of edges in *F* and in particular is replaced by a single vertex in $(G',P')$;
2. for each *s*-*e* path $Q\in S$ such that $s,e\in Y_t$ and $Q\cap E_t\ne \emptyset$, it holds that each path segment of *Q* outside of $Y_t$ begins and ends with a pair of edges in *I* and in particular is replaced by a pair of new terminals in $(G',P')$;
3. for each *s*-*e* path $Q\in S$ such that $\{s,e\}\cap Y_t=\{s\}$, it holds that the path segment of *Q* in $Y_t$ containing *s* ends with an edge in *L* and is replaced by a new terminal in $(G',P')$, and all other path segments of *Q* in $Y_t$ begin and end with a pair of edges in *F* and are hence replaced by single vertices in $(G',P')$.

From the above, we observe that *S* can be transformed into a solution $S'$ for $(G',P')$. The backward direction then follows by reversing the above observations; in particular, given a solution $S'$ for $(G',P')$, we use the fact that $\lambda$ is valid to expand $S'$ into a full solution *S* to (*G*, *P*).□

### The Dynamic Step

Let us begin by formalizing our aim for this subsection.

#### Lemma 7

*There is an algorithm which takes as input* (*G*, *P*) *along with a nice width*-*k* *tree-cut decomposition* $(T,{\mathcal {X}})$ *of* *G* *and a non-leaf node* $t\in V(T)$ *and* $D(t')$ *for every child* $t'$ *of* *t*, *runs in time* $(k|P|)^{\mathcal {O}(k^2)}$, *and outputs* *D*(*t*).

Finally, we introduce two simple reduction rules which will later help us reduce our problem to Simple EDP. The first ensures that two vertices of degree at most 2 are not adjacent to each other.

#### Reduction Rule 1

*Let* (*G*, *P*) *be an instance of* EDP *containing an edge* *ab* *between two vertices of degree at most* 2.

1. *If* *a* *is not a terminal, then contract* *ab* *and replace all occurrences of* *b* *in* *P* *by the new vertex;*
2. *If* $\{a,b\}\in P$, *then remove* $\{a,b\}$ *from* *P* *and remove the edge* *ab* *from* *G*;
3. *If* $\{a,b\}\not \in P$ *and each of* *a* *and* *b* *occurs in precisely one element of* *P*, *then delete the edge* *ab*;
4. *Otherwise, reject* (*G*, *P*).

#### Proof of Safeness

The safeness of the first three rules is straightforward. As for the fourth rule, let us consider the conditions for when it is applied. In particular, the fourth rule is only called if either *a* or *b* occurs in three terminal pairs, or if *a* occurs in at least one terminal pair and *b* in at least two but $\{a,b\}\not \in P$. Clearly, (*G*, *P*) is a **NO**-instance in either of these cases.□

The second reduction rule will allow us to replace thin nodes with data tables by small representatives; these representatives will only contain vertices of degree at most 2 adjacent to the original neighborhood of the thin node. For brevity and as a slight abuse of notation, we use the symbol $\mapsto$ to identify how the first element $\delta$ in a record partitions the edges in $E_t$.

#### Reduction Rule 2

*Let* *t* *be a thin node in* *V*(*T*) *with non-empty* *D*(*t*).

1. *If* $E_t=\{\{a,b\}\}$ *where* $a\in Y_t$ *and if*
  $((\{a,b\}\mapsto L'),\emptyset ,\emptyset ,\{s,\{a,b\}\})\in D(t)$ *for some* $s\in U_t$, *then delete* $Y_t{\setminus }\{s\}$ *and create the edge* *sb*;
  *otherwise,* $((\{a,b\}\mapsto U'),\emptyset ,\emptyset ,\emptyset )\in D(t)$ *and we delete* $Y_t$.
2. *If* $E_t=\{\{a,b\},\{c,d\}\}$ *where* $a,c\in Y_t$, $U_t=\emptyset$ *and if*
  $((\{a,b\}\mapsto F',\{c,d\}\mapsto F'),\emptyset ,\{\{a,b\},\{c,d\}\},\emptyset )\in D(t)$, *then delete* $Y_t$ *and create a new vertex* *v* *adjacent to* *b* *and* *d*; *else, if*
  $$((\{a,b\}\mapsto U',\{c,d\}\mapsto U'),\emptyset ,\emptyset ,\emptyset )\in D(t)$$, *then delete* $$Y_t$$;
  *otherwise,* $((\{a,b\}\mapsto I',\{c,d\}\mapsto I'),\{\{a,b\},\{c,d\}\},\emptyset ,\emptyset )\in D(t)$ *and we delete* $Y_t{\setminus }\{a,c\}$ *and add* $\{a,c\}$ *into the set* *P* *of terminals.*
3. *If* $E_t=\{\{a,b\},\{c,d\}\}$ *where* $a,c\in Y_t$, $U_t=\{s\}$ *and if*
  $((\{a,b\}\mapsto L', \{c,d\}\mapsto U'),\emptyset ,\emptyset ,\{s,\{a,b\}\})\in D(t)$ *and also* $((\{c,d\}\mapsto L', \{a,b\}\mapsto U'),\emptyset ,\emptyset ,\{s,\{c,d\}\})\in D(t)$, *then delete* $Y_t{\setminus }\{s\}$ *and make* *s* *adjacent to* *b* *and* *d*;
  *otherwise,* $((\{a,b\}\mapsto L', \{c,d\}\mapsto U'),\emptyset ,\emptyset ,\{s,\{a,b\}\})\in D(t)$ *and we delete* $Y_t{\setminus }\{s\}$ *and make* *s* *adjacent to* *b*.
4. *If* $E_t=\{\{a,b\},\{c,d\}\}$ *where* $a,c\in Y_t$, $U_t=\{s_1,s_2\}$ (*not necessarily* $s_1\ne s_2$) *and if*
  $((\{a,b\}\mapsto L',\{c,d\}\mapsto L'),\emptyset ,\emptyset ,\{\{s_1,\{a,b\}\},\{s_2,\{c,d\}\}\})\in D(t)$ *and* $((\{a,b\}\mapsto L',$ $\{c,d\}\mapsto L'),\emptyset ,\emptyset ,\{\{s_2,\{a,b\}\},\{s_1,\{c,d\}\}\})\in D(t)$, *then add a new vertex* $s'$ *adjacent to* *b* *and* *d*, *replace all occurrences of* $s_1$ *and* $s_2$ *in* *P* *by* $s'$, *and delete* $Y_t$;
  *otherwise*, $((\{a,b\}\mapsto L',\{c,d\}\mapsto L'),\emptyset ,\emptyset ,\{\{s_1,\{a,b\}\},\{s_2,\{c,d\}\}\})\in D(t)$ *and we delete* $Y_t{\setminus }\{s_1,s_2\}$, *and make* $s_1$ *adjacent to* *b* *and* $s_2$ *adjacent to* *d*.
5. *Otherwise*, (*G*, *P*) *is a* **NO** *-instance.*

The safeness of Reduction Rule 2 follows directly from the definition of *D*(*t*) (one simply needs to check each case separately) and hence we do not provide an explicit proof for each case. To provide intuition for Case 5., we note that:

- Case 1. captures the only two possible outcomes when $|E_t|=1$;
- Case 2. captures the only admissible outcomes when $|E_t|=2$ and $U_t=\emptyset$: the two edges in $E_t$ can either be used to connect a terminal pair outside of $G_t$, or remain unused, or used to connect a terminal pair inside of $G_t$;
- Case 3. captures the only admissible outcomes when $|E_t|=2$ and $U_t=\{s\}$: either it is possible to route *s* to either of the two edges in $E_t$, or only one of these two edges can be connected to *s* via an edge-disjoint path;
- Case 4. captures the only admissible outcomes when $|E_t|=2$ and $|U_t|=2$: either it is possible to route both of the unmatched terminals in $U_t$ to either of the two edges (in parallel), or parallel routing of both unmatched terminals to $E_t$ requires each terminal to be routed to precisely one fixed edge in $E_t$.

With Lemma 4 and Reduction Rules 1, 2 in hand, we have all we need to handle the dynamic step. It will be useful to recall the definitions of $A_t$ and $B_t$, and that $|A_t|\le 2k+1$.

#### Proof of Lemma 7

We begin by looping through all of the at most $4^k\cdot k!$ distinct records for *t*; for each such record $\lambda$, our task is to decide whether it is valid, i.e., whether $(G^{t,\lambda },P^{t,\lambda })$ is a **YES**-instance. On an intuitive level, our aim will now be to use branching and simplification in order to reduce the question of checking whether $\lambda$ is valid to an instance of Simple EDP.

In our first layer of branching, we will select a record from the data tables of each node in $A_t$. Formally, we say that a *record-set* is a mapping $\tau :t'\in A_t\mapsto \lambda _{t'}\in D(t')$. Note that the number of record-sets is upper-bounded by $(4^k\cdot k!)^{2k+1}$, and we will loop over all possible record-sets.

Next, for each record-set $\tau$, we will apply simplification to each node $t'\in A_t$ in accordance with $\tau (t')$, and recall that each vertex *v* created by this sequence of simplifications has degree at most 2. Next, we exhaustively apply Reduction Rule 1 to ensure that each such *v* is only adjacent to $(V(G){\setminus }Y_t)\cup X_t$. At this point, every vertex contained in a bag $X_{t'}$ for $t'\in A_t$ has degree at most 2 and is only adjacent to $X_t\cup (V(G){\setminus }Y_t)$.

Finally, we apply Reduction Rule 2 to replace each thin node by vertices of degree at most 2 adjacent to $X_t$. At this point, every vertex in $V(G^{t,\lambda }){\setminus }X_t$ is of degree at most 2 and only adjacent to $X_t$, and so $(G^{t,\lambda },P^{t,\lambda })$ is an instance of Simple EDP. All that is left is to invoke Lemma 4; if it is a **YES**-instance then we add $\lambda$ to *D*(*t*), and otherwise we do not.

The running time is upper bounded by the branching factor $(4^k\cdot k!)^{2k+1}$ times the time to apply our two reduction rules and the time required to solve the resulting Simple EDP instance. All in all, we obtain a running time of at most $k^{\mathcal {O}(k^2)}\cdot |P|^{\mathcal {O}(k^2)}=(k|P|)^{\mathcal {O}(k^2)}$.

We conclude the proof by arguing correctness. Assume $\lambda$ is a valid record. By Definition 3, this implies that $(G^{t,\lambda },P^{t,\lambda })$ admits a solution *S*. For each child $t'\in A_t$, *S* corresponds to some record $\lambda ^S_{t'}$ for *t*; consider now the branch in our algorithm which sets $\tau (t')=\lambda ^S_{t'}$. Then by Observation 1 it follows that each simplification carried out by the algorithm preserves the existence of a solution to $(G^{t,\lambda },P^{t,\lambda })$. Since both our reduction rules are safe, the instance of Simple EDP we obtain at the end of this branch must also be a **YES**-instance.

On the other hand, assume the algorithm adds a record $\lambda$ into $D_t$. This means that the resulting Simple EDP instance was a **YES**-instance. Then by the safeness of our reduction rules and by the second part of Observation 1, the instance obtained by reversing the reduction rules and simplifications was also a **YES**-instance; in particular $(G^{t,\lambda },P^{t,\lambda })$ is a **YES**-instance and so $\lambda$ is a valid record.□

We now have all the ingredients we need to prove our main result.

#### Theorem 2

EDP *can be solved in time at most* $\mathcal {O}(n^3)+k^{\mathcal {O}(k^2)} n^2+ (k|P|)^{\mathcal {O}(k^2)}n$, *where* *k* *is the tree-cut width of the input graph and* *n* *is the number of its vertices.*

#### Proof

We begin by invoking Theorem 1 to compute a tree-cut decomposition of *G* of width at most 2*k* and then converting it into a nice tree-cut decomposition (this takes time $k^{\mathcal {O}(k^2)} n^2$ and $\mathcal {O}(n^3)$, respectively). Afterwards, we use Lemma 6 to compute *D*(*t*) for each leaf of *T*, followed by a recursive leaf-to-root application of Lemma 7. Once we compute *D*(*r*) for the root *r* of *T*, we output **YES** if and only if $D(r)=\{(\emptyset , \emptyset , \emptyset , \emptyset )\}$.□

## Kernelizing EDP Parameterized by Feedback Edge Set

The goal of this section is to provide a fixed-parameter algorithm for EDP which exploits the structure of the input graph exclusively. While tree-cut width cannot be used to obtain such an algorithm, here we show that the feedback edge set number can. More specifically, we obtain a linear kernel for EDP parameterized by the feedback edge set number. Our kernel relies on the following two facts:

### Fact 1

*A minimum feedback edge set of a graph* *G* *can be obtained by deleting the edges of minimum spanning trees of all connected components of* *G*, *and hence can be computed in time* $\mathcal {O}(|E(G)|+|V(G)|)$.

### Fact 2

[14] EDP *can be solved in polynomial time when* *G* *is a forest.*

Consider an instance (*G*, *P*) of EDP and let $X\subseteq E(G)$ be a minimum feedback edge set *X*. Let *Y* be the set of all vertices incident to at least one edge from *X*. For the purposes of this section, it will be useful to view *P* as a multiset rather than a set. We begin with two simple reduction rules which allow us to remove some degree 2 vertices and all leaves disjoint from *Y*.

### Reduction Rule 3

*Let* $v,a,b\in V(G)$ *be such that* $N_G(v)=\{a,b\}$, $v\not \in Y$ *and* $ab \not \in E(G)$. *If* *v* *does not occur in any terminal pair in* *P*, *then delete* *v* *and add the edge* *ab* *into* *E*(*G*).

### Proof of Safeness

Observe that every solution to the original instance which uses an edge incident to *v* must contain a path which traverses through both *av* and *vb*, and after the reduction rule is applied one can simply replace these two edges in that path by *ab*. Any solution in the reduced instance can be similarly transformed into a solution to the original instance. Moreover, *X* clearly remains a feedback edge set in the reduced instance.□

### Reduction Rule 4

*Let* $v\in V(G)$ *be such that* $N_G(v)=\{w\}$. *Then:*

1. *if* *v* *occurs in no terminal pair in* *P*, *delete* *v* *from* *G*;
2. *if* *v* *occurs in precisely one terminal pair* $\{v,w\}$ *in* *P*, *delete* *v* *from* *G* *and delete* $\{v,w\}$ *from* *P*;
3. *if* *v* *occurs in precisely one terminal pair* $\{v,y\}$ *in* *P* *where* $y\ne w$, *delete* *v* *from* *G* *and replace* $\{v,y\}$ *in* *P* *by* $\{w,y\}$;
4. *if* *v* *occurs in at least two terminal pairs in* *P*, *reject* (*G*, *P*).

### Proof of Safeness

In the first case, it is easy to see that no path in the solution can contain *v*. For the second and third case, safeness follows by the fact that every path connecting *v* to its assigned terminal pair must use the edge *vw* and no other path can use *vw*. For the last case, simply observe that a leaf cannot appear in more than one edge-disjoint path.□

Observe that the exhaustive application of Reduction Rules 3 and 4 results in an instance (*H*, *L*) where every leaf lies in *Y*. Moreover, every vertex of degree 2 must lie in at least one terminal pair, or lie in *Y*, or be adjacent to a vertex in *Y* (since Reduction Rule 3 does not apply to a $C_3$). We now introduce a new rule and lemma which will help us deal with the potentially large number of vertices of degree 2 that occur in terminal pairs.

### Reduction Rule 5

*Let* $vw\in E(H)$ *be such that* $\{v,w\}\in L$. *Then remove* *vw* *from* *E*(*H*) (*and also from* *X*, *if it was in* *X*), *and remove* $\{v,w\}$ *from* *L*.

### Proof of Safeness

If the solution connects the terminal pair $\{v,w\}$ via the edge *vw*, the solution is preserved even after applying the rule. If the solution connects the pair using a different path, we can obtain an equivalent solution by instead connecting *v* to *w* via the edge *vw* and—if this edge was used to connect a different terminal pair—using the old *v*-*w* path as a replacement for that edge. Finally, if the reduced instance admits a solution, it is easy to see that the graph also had a solution before the application of the rule to delete *vw* and $\{v,w\}$.□

We can now prove the following for the instance $(H',L')$ obtained from (*H*, *L*) by exhaustively applying Reduction Rule 5.

### Lemma 8

*Let* $a,b,c\in V(H'){\setminus }Y$ *be three degree-2 vertices in* $H'$ *such that* $N(b)=\{a,c\}$. *Then* $(H',L')$ *is a* **NO***-instance.*

### Proof

By the exhaustive application of Reduction Rule 3, the vertex *b* must occur in at least one terminal pair. Moreover, since we have also exhaustively applied Reduction Rule 5, this terminal pair can be neither $\{b,a\}$ nor $\{b,c\}$. And since both *a* and *c* have degree 2, each of these must also occur in some terminal pair, say $\{a,a'\}$ and $\{c,c'\}$.

Now, to reach a contradiction let us consider a hypothetical solution *S* for $(H',L')$. Clearly *S* must contain an *a*-$a'$ path, and this path cannot start with the edge *ab* (since then it would have to continue with *bc*, preventing *b* from using any edge to reach its own terminal pair). By symmetry, *S* must also contain a *c*-$c'$ path which does not start with the edge *cb*. But now the only two vertices reachable by an edge-disjoint path from *b* are *a* and *c*, and we have argued that *b* has a terminal pair with a vertex different from *a* and *c*. Hence, we have reached a contradiction to the existence of *S*.□

At this point, we can prove that we have a linear kernel, as desired.

### Theorem 3

EDP *admits a linear kernel parameterized by the feedback edge set number of the input graph.*

### Proof

Let us consider the graph $(H',L')$ obtained by the exhaustive application of Reduction Rules 3–5. Since we have already established the safeness of these rules, it suffices to argue that the instance has size linear in |*Y*|. We now check if Lemma 8 applies—if yes then we reject, and otherwise we proceed knowing that *G* contains no path of 3 consecutive degree-2 vertices disjoint from *Y*.

Let us now consider the number of vertices in $H'$, which is the same as the number of vertices in the graph $\mathcal {Q}=H'-X$. By the exhaustive application of Reduction Rule 4, every leaf in $\mathcal {Q}$ lies in *Y* and hence in particular $\mathcal {Q}$ contains at most |*Y*| leaves. Consequently, the number of vertices of degree at least 3 is also upper-bounded by |*Y*|. It remains to bound the number of vertices of degree precisely 2 in $\mathcal {Q}$.

To this end, let *Z* be the union of *Y* with the set of all vertices of degree at least 3, and recall that $|Z|\le 2|Y|$. By the exhaustive application of Reduction Rule 3 and our use of Lemma 8, every vertex of degree 2 in $\mathcal {Q}$ must be a neighbor of at least one vertex in *Z*. The number of such vertices is upper-bounded by 2 times the number of edges of a tree with at most |*Z*| vertices, i.e., at most $2\cdot (2|Y|-1)$. We conclude that $\mathcal {Q}$ (and hence also $H'$) contains at most 6|*Y*| vertices. Moreover, $\mathcal {Q}$ contains at most 6|*Y*| edges and hence *G* contains at most $6|Y|+|X|\le 7|Y|$ edges.

To conclude the proof, it suffices to bound the size of $L'$. Here, we simply observe that a **YES**-instance cannot contain more terminal pairs than the number of edges in $H'$ (since terminal pairs always contain two distinct vertices), and so either $|L'|\le 7|Y|$ or we can correctly reject $(H',L')$.□

## Footnotes

1 For technical reasons, *F* will also store information about paths with unmatched terminals which use multiple edges in $E_t$—see Definition 4 later.
